# Effect of different sources of dietary zinc on sperm quality and oxidative parameters

**DOI:** 10.3389/fvets.2023.1134244

**Published:** 2023-06-20

**Authors:** Seyed Hamid Mousavi Esfiokhi, Mohammad Ali Norouzian, Abouzar Najafi

**Affiliations:** Department of Animal and Poultry Science, College of Aburaihan, University of Tehran, Tehran, Iran

**Keywords:** sperm, zinc, diet, antioxidant, motility

## Abstract

Zinc has a critical physiological role in sperm function. The purpose of this study was to investigate the effect of different sources of zinc on sperm quality. For this purpose, 18 Zandi lambs with an average weight of 32 ± 1.2 kg were subjected to three treatments in a completely randomized design. Experimental treatments include (1) control treatment of basal diet without zinc supplementation, (2) basal diet with 40 mg/kg of zinc supplementation from zinc sulfate source and (3) basal diet with 40 mg/kg of zinc supplementation with organic source. At the end of feeding period, lambs were slaughtered. To determine the effect of experimental treatments on sperm quality, the testes were transferred to the laboratory. After that, epididymal spermatozoa were evaluated for sperm motility parameters, abnormal morphology, viability, membrane functionality, malondialdehyde (MDA) and antioxidant activity [glutathione peroxidase (GPx), superoxide dismutase (SOD), total antioxidant capacity (TAC)], sperm concentration and testosterone level. Zinc sulfate administration decreased MDA levels compared to other treatments and increased GPx and TAC activity compared to the control (*P* < 0.05), although SOD activity was not affected by any supplementation. Also, the use of zinc sulfate supplementation increased the percentage of total and progressive motility compared to the control group (*P* < 0.05). Membrane integrity and sperm viability were also affected by zinc sulfate supplementation (*P* < 0.05). Therefore, the results of this study showed that the use of zinc sulfate, can improve sperm motility and survival indices and its antioxidant capacity.

## 1. Introduction

Zinc is one of the essential nutrients for animals and humans which is required for many antioxidant functions, growth, reproduction and safety (1). The second-most prevalent mineral in the body, zinc has crucial roles in development, immunological response, reproduction, gene expression, and the wool production (2). Zinc supplements are divided into mineral resources (such as sulfate and zinc oxide) and organic sources (such as zinc-methionine, zinc-protein and zinc-lysine). Zinc is essential for numerous physiological functions in animals, plants and humans (3). Moreover, zinc has a critical physiological role in the function of sperm cells, which includes the effect on their motility and maintenance of their natural morphology (4, 5). Zinc is vital in maintaining sperm characteristics (4, 5). It has been shown that zinc deficiency impairs spermatogenesis in rats and causes atrophy of the seminiferous tubule. Consumption of zinc supplements in buffalo has improved reproductive performance (6). It has been reported that supplementation of zinc improved reproductive performance, lamb production, and health (7). Zinc is one of the contributing factors in the antioxidant system which causes dismutation of superoxide anion due to aerobic metabolism and converts it to hydrogen peroxide. In addition, it acts as a cofactor for coping with oxidative stress (8).

Zinc in semen is directly related to normal morphology, viability, and directly related to sperm motility (9). Elements such as selenium and zinc are essential for sperm production and testicular growth (10). Zinc is an antioxidant element and has, protective role against free radicals (11). Adding zinc supplement to the diet eliminates many side effects of free radicals (12). The quality of the sperm and the fertility of rams are influenced by a number of variables, including breed, age, season, and nutritional management (13).

Zinc supplementation improves sperm motility in male buffaloes (14). Sperm membrane integrity is essential not only for sperm metabolism but also for favorable changes in characteristics of male and female gametes (15). Insufficient intake of Zn impairs the antioxidant defense system (16). In another study, the addition of zinc nanoparticles to bovine sperm after freezing-thawing process increased membrane integrity and mitochondrial activity of sperm compared to the control group (17). Zinc in human semen plays an essential role in the physiological function of sperm, and its deficiency results in reduced levels of low sperm quality and reduced chances of fertility (10, 18). Antioxidants protect sperm from ROS production, DNA damage, plasma and mitochondrial membrane damage, reduced motility and viability, untimely maturation, damage from the freeze-thaw process, improve quality sperm, and increase motility, viability and fertility of sperm (19–21).

Moreover, to our knowledge, there are no reports about various zinc supplements on ram sperm quality parameters. Therefore, the objective of this study was to evaluate the effect of various zinc supplements on sperm quality parameters. We evaluated the conventional sperm parameters such as motility and viability and the presence and direct markers of the oxidative status, such as malondialdehyde (MDA) levels and antioxidants GPx, SOD and TAC (total antioxidant capacity).

## 2. Materials and methods

### 2.1. Chemical

Chemicals were obtained from Merck (Darmstadt, Germany) and Sigma-Aldrich Company (St. Louis, MO).

### 2.2. Animals and semen collection

During the experiment, in breeding season 18 lambs with a mean age of 120 ± 10 days and an average weight of 32 ± 1.2 kg were randomly divided into three groups. Eighteen individual cages were used to keep the lambs in the experimental period. Experimental treatments include (1) control treatment of basal diet without zinc supplementation, (2) basal diet with 40 mg/kg of zinc supplementation from zinc sulfate source and (3) basal diet with 40 mg/kg of zinc supplementation with its organic origin. All experiments were performed in the University of Tehran, Iran.

The diets were formulated in accordance with the NRC (22) recommendations and animals were fed a total mixed ration ad libitum. Every lamb was housed in a separate pen with a cement floor and access to individual feeding and watering. At 08 and 16 hours, feed was provided twice daily in quantities that permitted 10% refusal. The amount of supplements was adjusted daily based on DMI of individual lambs. The current experiment lasted for 85 days. At the end of feeding period, lambs were slaughtered following a 12 h feed removal, based on the standard slaughter protocol in experimental abattoir of the farm of college of agriculture. To determine the effect of experimental treatments on sperm quality of experimental lambs, the testes were transferred to the laboratory. The tunica vaginalis of the testicles was removed in the laboratory. Epididymides with vas deferens were detached from the testis. A 35 mm petri dish was used to hold each cauda epididymis after it had been dissected free and rinsed with 0.9% saline. With the use of forceps and a scalpel, many incisions were made in the tubuli of the cauda epididymides and immersed in of tris buffer for sperm migration for 10 min. Collected sperm were washed once by centrifugation (5 minute) with tris base extender at 400 g (23). The sperm concentration was evaluated by a hemocytometer (24).

### 2.3. Testosterone assessment

Blood samples were taken from wing vein into EDTA anticoagulant. The tubes were centrifuged (10 min, 1500 g) and plasma were separated and stored at −20°C until assessment. Plasma testosterone level was evaluated by ELISA using commercial ELISA kit.

### 2.4. Sperm motility

Assessment of sperm motility was conducted using computer assisted sperm analyzer (CASA V 12.2; Hamilton Thorne Biosciences, Beverly, MA, USA). For each sample, at least 200 sperm were examined using normal procedures (37°C, 60 frames/s). The motility parameters evaluated for each sperm contained the curvilinear velocity (VCL, μm/s), the average path velocity (VAP, μm/s), the straight-line velocity (VSL, μm/s), the straightness (STR, %; VSL/VAP), the linearity (LIN, %; VSL/VCL), the beat cross frequency (BCF, Hz) and the amplitude of lateral head displacement (ALH, μm) (25).

### 2.5. Sperm abnormalities

To assess sperm morphology, 15 μl of semen was placed in tubes containing 1 ml of Hancock solution (426 mM sodium, 21.4 mM formalin, 304.29 mM Na_2_HPO_4_ and 99.42 mM K_2_HPO_4_) (26). A minimum of 200 spermatozoa were analyzed for morphologic abnormalities (head abnormalities, detached heads, abnormal mid-pieces and tail defects) using a phase contrast microscope (Labomed LX400; Labomed Inc., Culver City, CA) ( × 400 magnification).

### 2.6. Sperm viability

By staining with nigrosin-eosin, the vitality of the sperm was evaluated (27). By combining 10 μl of semen with 10 μl of stain on a heated slide and quickly spreading it with a second slide, sperm smears were created. A phase-contrast microscope (Labomed LX400; Labomed Inc., Culver City, CA) was used to examine 200 cells for viability ( × 400). Only unstained sperm heads (white color) were regarded viable, whereas spermatozoa that showed either partial or total purple staining were regarded non-viable.

### 2.7. Plasma membrane integrity test (HOST)

For evaluating this parameter, 10 μl of semen sample were gently mixed with 100 μl of hyposmotic solution and incubated for 30 minutes in a warm water bath (37°C) (28). Afterwards, 5 μl of the sample was placed on a preheated slide and covered with a slide. The slide was then placed on the hot plate of a contrast phase microscope (Labomed LX400; Labomed Inc., Culver City, CA). Two hundred sperm were counted with a magnification of 400 ×.

### 2.8. MDA concentration

The thiobarbituric acid (TBA) reaction was used to determine the MDA concentration as a marker of the lipid peroxidation in the semen samples (28). Briefly, 1 ml of trichloroacetic acid was added to each sample. Centrifugation was performed at 1200 × g for 10 min and then 1 mL thiobarbituric acid (0.375%) was added to supernatant. For 10 minutes, the tubes were incubated in boiling water. After cooling to room temperature, the samples were examined with a UV/Visible spectrophotometer (T80 UV/VIS PJ Instruments Ltd, UK) at 532 nm.

### 2.9. TAC, GPx, and SOD determination

The antioxidant system was evaluated by defining the TAC, and GPx and SOD activities (29). They were determined spectrophotometrically by Olympus AU 400 automatic biochemistry analyzer (Olympus, Tokyo, Japan), converting absorbance to specific units with a calibration curve. TAC was calculated by including the kit's reactivity, measuring absorbance at 600 nm, and converting absorbance to mmol/l. In the presence of oxidized glutathione (cumene hydroperoxide) and NADPH, GPx was assessed. The oxidized glutathione was subsequently reduced by GPx while simultaneously oxidizing NADPH to NADP+, resulting in a reduction in absorbance at 340 nm. The degree of inhibition of the oxidation of 2-(4-iodophenyl)-3-(4-nitrophenol)-5-phenyltetrazoliumchloride (INT) to the red formazan dye by superoxide radicals (generated by a xanthine/xanthine oxidase system) was used to determine SOD. Under the test conditions, one unit of SOD prevents a 50% drop in INT. At 505 nm, the absorbance was measured.

### 2.10. Statistical analysis

SAS software (version 9.1) was selected to analyze the data. For checking the normality of the data, the Shapiro-Wilk test was used. The effects of the treatments were then tested using linear mixed-effect models [PROC MIXED; (30)]. Tukey's test was selected for comparing treatments when the models were significant. The significance level was P < 0.05. Results are presented as mean ± SEM.

## 3. Results

Motility and velocity parameters of ram treated with a different form of zinc are presented in Table 1. Total motility was higher in the zinc sulfate treatment (p < 0.05) compared to other treatments. Also, PM showed higher motility (P < 0.05) in the zinc sulfate treatment than others. Motion parameters (VAP, VSL, VCL, ALH, LIN, BCF and STR) showed numerically higher values compared to organic and control supplementation, while there was no significant difference.

**Table 1:** Effect of zinc supplementation on semen motility parameters in Zandi lambs analyzed by CASA.

**Parameters**	**Treatments**				***P* value**
	**Control**	**Zinc sulfate**	**Zinc organic**	**SEM**	
TM (%)	79.66^ab^	90.12^a^	82.33^b^	1.87	0.01
PM (%)	44.66^b^	57.23^a^	47.66^b^	2.09	0.01
VAP (μm/s)	52.25	62.67	56.37	2.70	0.08
VSL (μm/s)	39.68	47.82	42.27	2.32	0.11
VCL (μm/s)	97.64	105.22	99.23	3.87	0.40
LIN (%)	40.59	45.62	42.78	2.53	0.42
STR (%)	76.23	76.41	75.12	3.83	0.96
ALH (μm)	3.15	3.17	3.25	0.24	0.95
BCF (Hz)	16.43	17.44	16.49	1.38	0.84

TM, Total motility; PM, Progressive motility; VSL, straight-line velocity; VAP, Average path velocity; VCL, curvilinear velocity; LIN, Linearity; STR, Straightness; ALH, Mean amplitude of the lateral head displacement; BCF, Mean of the beat cross frequency. Different superscripts within the same row indicate differences among groups (P < 0.05).

The use of the 40 mg/kg dry matter of zinc sulfate improved GPx, testosterone level and TAC compared to the control group. SOD activity and sperm concentration were not affected by any treatment (Table 2). The MDA level was found to be lower (P < 0.05) in the 40 mg/kg dry matter of zinc sulfate in comparison to the control group.

**Table 2:** Effect of zinc supplementation on malondialdehyde (MDA) concentration, glutathione peroxidase (GPx) and superoxide dismutase (SOD) activity, total antioxidant capacity (TAC), sperm concentration and testosterone level of Zandi lambs.

	**Parameters**					
**Treatments**	**MDA (nmol/l)**	**GPX (U/mg protein)**	**SOD (u/mg)**	**TAC (mmol/l)**	**Sperm concentration [sperm/mL ( × 10^9^)]**	**Testosterone (ng/mL)**
Control	4.14^a^	46.85^ab^	106.03	1.14^b^	2.76	6.12^b^
Zinc sulfate	2.70^b^	57.83^a^	118.57	2.16^a^	3.24	7.15^a^
Zinc organic	4.01^a^	48.05^b^	107.47	1.19^b^	2.95	6.21^b^
SEM	0.26	1.72	7.01	0.12	0.24	0.22
P Value	0.01	0.007	0.43	0.001	0.43	0.0002

Different superscripts within the same column indicate differences among groups (P < 0.05).

Data on abnormal sperm structure, membrane integrity, and viability are presented in Table 3. Sperm viability and membrane integrity were higher in the group with 40 mg/kg dry matter of zinc sulfate and in the organic zinc group compared to the control group. When compared to the control group, there was no significant difference in abnormal morphology after zinc addition.

**Table 3:** Effect of zinc supplementation on viability (eosin/negrosin), membrane integrity, abnormal forms of Zandi lambs.

	**Parameters**		
**Treatments**	**Membrane integrity (%)**	**Abnormal forms (%)**	**Viability (%)**
Control	74.01^b^	9.01	81.12^ab^
Zinc sulfate	87.52^a^	7.32	92.50^a^
Zinc organic	77.41^b^	8.56	84.48^b^
SEM	1.96	0.68	2.06
P Value	0.006	0.27	0.02

Different superscripts within the same column indicate differences among groups (P < 0.05).

## 4. Discussion

Studies have shown that zinc is very important in sperm membrane stability, viability, motility, and morphology. Optimal zinc plasma concentrations increase sperm concentration, motility, and antioxidant activity (31). On the other hand, excessive zinc consumption can lead to testicular destruction and loss of sperm motility in mice (32). The role of zinc in the production of vital enzymes has been proven to be an essential component of semen. Zinc may also indirectly affect the secretion of gonadotropic hormones through the pituitary gland (33). The lack of effect of zinc supplementation on sperm concentration in this test and other reports is due to the insufficient amount of zinc in the basal diet.

Rahman et al. (34) in their study on 16 male goats showed that the use of zinc supplementation as zinc sulfate significantly increased the amount of semen volume and sperm motility compared to the control group, but no significant relationship between sperm concentration and percentage of live sperm Zinc was observed in the receiving group and the control group.

Afifi et al. (35) evaluated the effect of zinc oxide nanoparticles on the activity of some antioxidant enzymes and sperm characteristics in diabetic mice. Their study showed that zinc oxide nanoparticles increase the activity of some antioxidant enzymes, including superoxide dismutase. They observed an increase in sperm motility and concentration compared to the control group. Similar to these results, zinc supplementation significantly increased the motility sperm (5). Similar studies have been reported in rabbits (36). Zinc sulfate supplementation with a level of 17.5 micromoles had an increasing effect on the percentage of total sperm (37). In a study on Barbari buck semen, volume, sperm motility, sperm viability and survival, and acrosome health improved (38). However, in some studies, contradictory results have been seen. For example, in a study conducted on ram sperm, the results showed that zinc sulfate has no significant effect on sperm motility (39). Jahanbin et al. (17) did not observe any significant effect on sperm motility characteristics by adding high levels of zinc in the form of zinc nanoparticles to the semen of Holstein bulls. Similar to the results of this study, zinc supplementation caused a significant increase in sperm survival (16). Kumar et al. (40) observed that zinc consumption in bulls in the form of zinc sulfate and zinc propionate increased sperm viability. Similar to the results of the present study, the use of zinc sulfate supplements has significantly increased the health of sperm membranes. Omu et al. (41) also reported that zinc supplementation improves sperm membrane health in men. In addition, KendallandTelfer (42) reported that zinc intake can improve sperm membrane health in sheep sperm. Similar to our findings, Jahanbin et al. (17) observed that membrane integrity was improved by adding zinc nanoparticles compared to control in bovine sperm. Changes in membrane integrity caused by cold shock damage, ice formation, and osmotic stress are the key factors decreasing the motility and viability of frozen sperm (43), and reactive oxygen species have been reported (types of active oxygen, which leads to oxidation) (29). Therefore, improving antioxidant indices by consuming zinc can lead to their improvements. In contrast, Orzołek et al. (44) found that turkey ejaculates enriched with Zn and kept for 48 hours had a few lower sperm parameters, including total and progressive motility and mitochondrial membrane potential. Perhaps the use of a high concentration of Zn in the study by Orzołek et al. (44) caused the reduction in sperm quality.

There is a significant relationship between testosterone and plasma zinc concentrations, so zinc supply is essential for sperm function (45). Zinc is essential for the considerable cell division required for semen production because it plays a significant role in the metabolism of nucleic acids and proteins (46). Zinc supplementation increases daily sperm production and lowers the percentage of defective spermatozoa (40, 47). In a study of the effect of organic and mineral resources on the concentration of organic hormones quantitative and qualitative traits of semen in male calves were examined for 6 months. By adding zinc to the diet, quantitative and qualitative traits of semen such as volume, motility, density and percentage of live sperm and testosterone concentration increased significantly (40). The effect of organic zinc and selenium supplementation was investigated in a study on sheep and contrary to our results, malondialdehyde concentration was not affected by experimental groups (48).

Rahman et al. (34) also reported that consumption of up to 200 parts per million zinc in the form of zinc sulfate for three months did not effect increasing sperm concentration in goats. In many studies, zinc sources as an oral supplement in the diet have an antioxidant role. It improves sperm quality indicators by stabilizing the antioxidant role in removing free radicals from the environment (49). Although in this experiment, zinc supplementation did not affect sperm concentration, but in an experiment, the use of organic and inorganic zinc supplementation in a bull diet improved the quantitative characteristics of ejaculation volume, sperm concentration and sperm count and sperm viability (40). It has been demonstrated that zinc plays a crucial function in the activity of many enzyme systems, and the role of this element in spermatogenesis and their maturation is well established (50). Zinc deficiency has severe effects on reproductive growth and yield of rams (51). Minimum dietary zinc concentrations of 30 mg/kg dry matter are recommended to prevent adverse effects on reproductive performance (51).

## 5. Conclusion

In the present study, supplementation of 40 mg/kg dry Zinc sulfate increased GPx and TAC activity while malondialdehyde (MDA) decreased. In addition, there was a higher percentage of total and progressive motility, membrane integrity and sperm viability.

## Data availability statement

The raw data supporting the conclusions of this article will be made available by the authors, without undue reservation.

## Ethics statement

The animal study was reviewed and approved by Department of Animal and Poultry Science, College of Aburaihan, University of Tehran, Tehran, Iran. Written informed consent was obtained from the owners for the participation of their animals in this study.

## Author contributions

MN designed and coordinated the study and collected data. AN and SM carried out the experiments and collected data. MN, AN, and SM wrote the manuscript and analyzed the data. All authors contributed to the article and approved the submitted version.
